# Tenascin-C as a predictor of delayed graft function after kidney transplantation

**DOI:** 10.3389/fimmu.2026.1682962

**Published:** 2026-02-17

**Authors:** Ziyan Yan, Yibin Wang, Yuchen Wang, Shengquan Wu, Wenli Zeng, Jialiang Hui, Jian Xu, Renfei Xia, Yun Miao

**Affiliations:** Department of Transplantation, Nanfang Hospital, Southern Medical University, Guangzhou, China

**Keywords:** acute kidney injury, biomarker, delayed graft function, kidney transplantation, Tenascin-C

## Abstract

**Background:**

Incidence of delayed graft function (DGF) increases due to the decline in donor kidney quality and the increased use of marginal allografts, while the promising biomarkers for early DGF prediction are lacking. Previous analyses showed that Tenascin-C (TNC) was associated with acute kidney injury; however, its correlation with DGF is unclear. This study aimed to evaluate the ability of TNC to predict DGF.

**Methods:**

This prospective study included 36 perioperative kidney transplant recipients. Serum and urine samples were collected at regular intervals before and during the 10 days after transplantation to measure TNC and other conventional biomarkers. Pre-implantation graft renal biopsies were analyzed using Remuzzi and TNC staining scores. These data were then combined with clinical risk factors to construct a DGF prediction model.

**Results:**

In recipients with DGF, sTNC levels peaked on postoperative day 4, and were associated with increased risk of composite events (DGF and rehospitalization). uTNC levels were significantly higher in recipients without DGF, peaking at 8 hours postoperatively. sTNC levels at postoperative day 4 and TNC immunohistochemical scores were identified as independent risk factors for DGF. Incorporating the above two factors into a model comprising recipient age, cholesterol levels, donor cold ischemia time, and surgery duration significantly improved its ability to predict DGF, with the area under the curve increasing from 0.6790 to 0.9321.

**Conclusion:**

This study highlights the TNC levels in perioperative kidney transplant recipients and their correlation with DGF. sTNC levels and TNC immunohistochemical staining scores may serve as potential biomarker predicting DGF.

## 1 Introduction

Delayed graft function (DGF) is a common early complication following kidney transplantation and is associated with adverse prognostic events such as prolonged postoperative hospitalization, acute rejection, chronic rejection, and allograft loss (1, 2). Patients with DGF have an approximately 1.5 times higher risk of renal allograft loss within 5 years than those without DGF (1). In the era of expanded criteria donors, DGF incidence is estimated to occur in 4–10% of living donor transplant recipients and 19–70% of deceased donor transplant recipients worldwide (3, 4).

The pathophysiology of DGF primarily involves ischemia-reperfusion injury (IRI), inflammation, endothelial dysfunction, immune responses, and tubular cell injury, among others (5, 6). Several risk factors have been identified in the onset of DGF, including donor-related (age, ischemia time and kidney donor profile index), recipient-related (IRI, matching status, and dialysis duration), and perioperative (surgery duration) factors (1, 5, 6). DGF diagnosis is based on clinical symptoms, and its pathogenesis, severity, and prognosis exhibit significant heterogeneity (7, 8). However, conventional methods for evaluating renal allografts, such as biopsy, serum creatinine (Scr) measurement, estimated glomerular filtration rate (eGFR), and proteinuria, are usually ineffective and nonspecific to predict the early occurrence and progression of DGF. Consequently, early biomarkers of DGF are urgently needed to promptly assist nephrologists in selecting the best treatment option.

Tenascin-C (TNC) is a large extracellular matrix glycoprotein with a monomeric molecular weight of approximately 180–400 kDa, which typically assembles into a hexamer *in vivo* (9–11). Its synthesis is tightly regulated, with widespread and restricted distribution in embryonic and adult tissues, respectively. TNC is expressed *de novo* during wound healing or in pathological conditions, including inflammation, tumors, and tissue injury repair (12). They are produced in the endoplasmic reticulum in a caveolin-1-dependent manner, secreted via exosomes, and widely distributed through the body to propagate inflammatory signals (13, 14). TNC promotes Wnt/β-catenin signaling, which is typically suppressed in adults but is reactivated during organ and tissue injury and regeneration (15, 16).

Research on TNC in the context of kidney transplantation is currently limited, with only one study reporting a correlation between increased TNC expression and acute renal allograft rejection (17). Moreover, evidence supporting the use of TNC as a biomarker for early graft dysfunction is lacking. Since DGF manifests as acute kidney injury (AKI) after kidney transplantation, our previous research identified TNC as a key mediator in acute kidney injury (AKI) and renal fibrosis, demonstrating significantly elevated levels in damaged kidneys that correlate with the severity of renal dysfunction and fibrosis (15, 16, 18–20). TNC is specifically induced *de novo* at injury sites and localizes in the renal interstitium, creating a favorable microenvironment for tubular repair and regeneration by promoting Wnt/β-catenin signaling after AKI (16). However, sustained overexpression of TNC can exacerbate kidney injury by interacting with specific cell surface receptors such as integrins and toll-like receptors and promoting inflammation (21, 22). Utilizing the pathological expression and function of TNC is becoming a promising strategy for developing new diagnostic, therapeutic, and bioengineering tools.

We hypothesized that TNC levels could serve as a non-invasive biomarker for the early prediction of DGF. Accordingly, this prospective study analyzed perioperative TNC levels in serum and urine (sTNC and uTNC), alongside other injury biomarkers in patients undergoing kidney transplantation. TNC was also combined with clinical risk factors to construct a predictive model for DGF to guide precise diagnosis and treatment.

## 2 Materials and methods

### 2.1 Ethics statement

This study was approved by the Ethic Committee of Nanfang Hospital, Southern Medical University (NFEC-2020-044), and was performed following the principles of the Declaration of Helsinki. No organs were procured from prisoners, and all organ donations were voluntary. Written informed consent was provided by all patients involved in our study.

### 2.2 Patients and procedures

Eligible participants were patients aged 18–75 years old who received their first kidney transplantation from June 2022 to December 2023 at Department of Organ Transplantation, Nanfang Hospital, Southern Medical University. Exclusion criteria included exposure to nephrotoxin (i.e., contrast media, aminoglycoside antibiotics, vancomycin, and nonsteroidal anti-inflammatory drugs except aspirin) within 4 weeks before surgery and preexisting advanced urinary tract infection. Each patient voluntarily participated in the study and signed a written informed consent. All kidney allografts were from donation after brain death (DBD) or after cardiac death (DCD) donors. We collected spot blood samples before operation and collected spot urine and blood samples at frequent intervals for 10 days after kidney transplantation. Urine samples were collected every 4 hours for the first 8 hours after kidney transplantation, and then every 24 hours for the first 4 days after surgery. Blood samples were obtained at 8 hours after surgery, and then every 24 hours for the first 4 days after surgery. Urine and blood samples were collected every 48 hours from the fourth to the tenth day after surgery. The urine and blood samples were centrifuged at 3000 g for 10 minutes and the supernatants were stored at -80 °C. DGF was defined primarily based on Scr levels and the need for dialysis, specifically by dialysis within 7 days of the transplant, the failure of Scr levels to decrease to 400 μmol/L, or a daily reduction in Scr levels of less than 10% for three consecutive days (7).

### 2.3 TNC enzyme-linked immunosorbent assay

Urinary and plasma Tenascin-C level were measured by Human Tenascin-C Assay Kit was purchased from the Immuno-Biological Laboratories (IBL) (#27767; IBL Company, Gunma, Japan) according to the manufacturer’s instructions. This assay employs the quantitative sandwich enzyme immunoassay technique, which can detect TNC high molecular weight variants including the FNIII-B domain. Human urinary and serum TNC were measured according to the assay procedures specified by the manufacturer. Urinary creatinine was measured using an automatic biochemical analyzer (AU480; Olympus, Tokyo, Japan). Urinary TNC levels were calculated after normalization with urinary creatinine and expressed as nanograms per milligram creatinine. Serum TNC levels were expressed as nanograms per milliliter.

### 2.4 HLA typing

High-resolution typing of HLA-A/B/C/DRB1/DQB1/DPB1 loci was performed using GenDx NGSgo kits and analyzed with NGSengine software.

### 2.5 Histology and immunohistochemical staining

Paraffin-embedded the pre-implantation graft renal biopsies were prepared by a routine procedure. The sections were stained with Hematoxylin & Eosin (HE), Periodic Acid-Schiff (PAS), and stained for TNC (1:250, ab108930, Abcam). The staining intensity of all sections was observed, scored, and recorded under a microscope by two investigators in a double-blind manner. Biopsies were evaluated for glomerulosclerosis, tubular atrophy, interstitial fibrosis, and arteriolar narrowing, and were used to calculate the Remuzzi score. Each feature was graded from 0 to 3 based on severity, with higher scores indicating a more severe pathology. The total score was the sum of the individual scores, with a Remuzzi score ≥4 considered high. The immunostaining results for TNC were measured based on the proportion and intensity of positive staining in the renal cortical interstitium. Two experienced pathologists independently evaluated TNC staining using the immunoreactivity score (IRS). Staining intensity was scored as follows: 0 (no staining); 1 (light yellow, weak); 2 (yellow, moderate); and 3 (brown, strong). The extent of positive staining was scored as follows: 0 (0%), 1 (0–10%), 2 (10–50%), and 3 (>50%). The final IRS was generated by multiplying the scores for staining intensity by those for the extent of positive staining.

### 2.6 Statistical analyses

GraphPad Prism 9.5.1 was used for analysis. Categorical variables were compared using chi-square or Fisher’s exact test; continuous variables with t-tests or ANOVA. Repeated-measures ANOVA assessed trends over time. Logistic regression was used to estimate predictive performance. A two-sided p-value <0.05 was considered significant.

## 3 Results

### 3.1 Cohort description

This study included 36 adult patients (24 male and 12 female) receiving kidney transplants. The 36 renal allografts were obtained from 19 deceased donors (16 male and 3 female), with an average age of 38.9 ± 9.9 years. Of the 36 allografts, 4 were obtained by donation after circulatory death, and the remaining 32 were obtained by donation after brain death.

In the majority of recipients (77.78%, 28/36), the immunoinduction therapy regimen consisted of “ basiliximab + methylprednisolone”, and the postoperative immunomaintenance regimen for all recipients was “tacrolimus + mycophenolate mofetil + prednisolone”. A total of 18 recipients (50%) developed DGF. Recipients with and without DGF had similar baseline characteristics, including sex (male/all, 11/18 vs. 13/18, p=0.725), body mass index (21.7 ± 3.8 vs. 21.9 ± 2.4 kg/m², p=0.840), and the presence of the underlying diseases hypertension (p>0.999), diabetes (p=0.658), and hepatitis B (p>0.999). Additionally, recipients with and without DGF had similar donor and matching characteristics, including donor age (39.4 ± 9.7 vs. 38.3 ± 10.3 years, p=0.741), sex (male/female, 14/4 vs. 16/2, p=0.658), HLA matching (p=0.776), and Remuzzi score (p=0.943).

Although no significant differences were observed in baseline characteristics between recipients with and without DGF, recipients with DGF tended to be older (43.4 ± 10.4 vs. 40.1 ± 12.5 years) and to have higher total cholesterol levels (5.0 ± 3.9 vs. 4.1 ± 1.2 mmol/L) than those without DGF. Additionally, the duration of surgery tended to be longer in recipients with DGF (4.2 ± 1.0 vs. 3.7 ± 0.5 h). The incidence of proteinuria at 3 months postoperatively and rehospitalization within 6 months postoperatively were higher in patients with DGF than in those without DGF (56% vs. 33%; 39% vs. 22%), although again these differences did not reach significance. At 3 and 6 months postoperatively, recipients with DGF generally had lower Scr levels than patients without DGF, as presented in **Table 1**.

**Table 1:** Description of donors and recipients cohort after kidney transplantation.

Characteristic	All (n=36)	Not-DGF (n=18)	DGF (n=18)	p value
Recipients				
Age, yr	41.7 ± 11.5	40.1 ± 12.5	43.4 ± 10.4	0.390
Men, n (%)	24 (66)	13 (72)	11 (61)	0.725
Hypertension, n (%)	32 (89)	16 (89)	16 (89)	>0.999
Diabetes mellitus, n (%)	6 (17)	4 (22)	2 (11)	0.658
HBV or Carrier, n (%)	7 (19)	3 (17)	4 (22)	>0.999
BMI, kg/m^2^	21.8 ± 3.2	21.9 ± 2.4	21.7 ± 3.8	0.840
Cholesterol, mmol/L	4.6 ± 2.9	4.1 ± 1.2	5.0 ± 3.9	0.378
Donors and typing				
Age, yr	38.9 ± 9.9	38.3 ± 10.3	39.4 ± 9.7	0.741
Men, n (%)	30 (83)	16 (89)	14 (78)	0.658
DBD, n (%)	32 (89)	17 (94)	15 (83)	0.603
Warm Ischemia, n (%)	4 (11)	1 (6)	3 (17)	0.603
CIT, hr	4.3 ± 2.2	4.6 ± 2.4	4.1 ± 2.0	0.499
HLA typing	2.1 ± 1.1	2.1 ± 1.2	2.0 ± 1.1	0.776
HLA typing >2/6, n (%)	10 (28)	4 (22)	6 (33)	0.711
Remuzii score	2 (1-4.5)	2 (1-5)	2.5 (1-4)	0.943
Scr, μmol/L	105.5 (80.8-148.0)	95.0 (77.0-136.5)	108.0 (88.0-170.5)	0.458
Urine Creatinine, mmol/L	4.3 (1.2-9.6)	4.7 (1.8-9.6)	1.8 (0.8-9.6)	0.256
Remuzzi with high score, n (%)	13 (36)	7 (39)	6 (33)	>0.999
IIR				
MP, n (%)	2 (6)	1 (6)	1 (6)	0.755
BAM+MP, n (%)	28 (78)	14 (78)	14 (78)	
ATG+MP, n (%)	5 (14)	2 (11)	3 (17)	
BAM+ATG+MP, n (%)	1 (3)	1 (6)	0	
Surgery				
Surgical duration, hr	3.9 ± 0.8	3.7 ± 0.5	4.2 ± 1.0	0.061
Hospital admission				
Length of hospital stay, day	15.0 ± 3.8	14.7 ± 3.7	15.3 ± 4.0	0.670
Hospital readmission within six ms, n (%)	11 (31)	4 (22)	7 (39)	0.471
Others				
Scr_3 ms post KT, μmol/L	170.1 ± 128.3	132.4 ± 31.5	205.8 ± 171.0	0.091
Scr_6 ms post KT, μmol/L	171.6 ± 138.1	139.4 ± 74.1	202.0 ± 175.9	0.184
Proteinuria_3 ms post KT, n (%)	16 (44)	6 (33)	10 (56)	0.315
Proteinuria_6 ms post KT, n (%)	20 (56)	9 (50)	11 (61)	0.738
HGB_3 ms post KT, g/L	127.3 ± 18.1	131.1 ± 18.4	123.4 ± 17.4	0.208
HGB_6 ms post KT, g/L	132.4 ± 25.0	138.2 ± 25.2	126.5 ± 24.1	0.162

BMI, Body mass index; CHOL, cholesterol; DBD, donation after brain death; CIT, cold ischemia time; HLA, human leukocyte antigen; MP, methylprednisolone; ATG, rabbit anti-human thymocyte immunoglobulin; BAM, Basiliximab; IIR, immune induction regimen.

### 3.2 Serum and urine TNC levels as predictors of DGF

**Figure 1:**
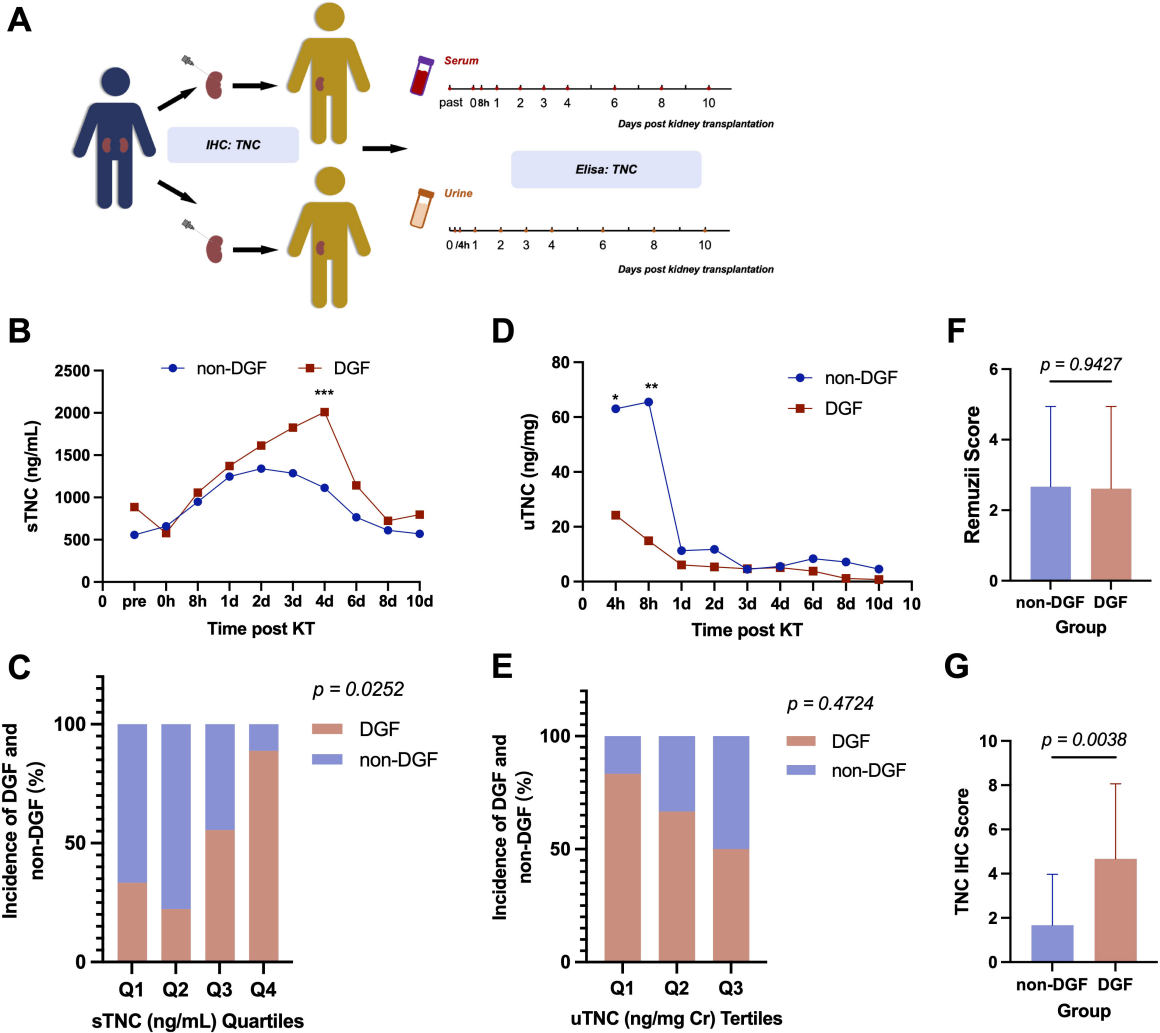
**(A)** Workflow in the study. **(B)** Elisa results of sTNC level during perioperative period of kidney transplantation. **(C)** Quartiles of sTNC on the 4th day after KT showed a graded relationship with the incidence of DGF. **(D)** Elisa results of uTNC level during perioperative period of kidney transplantation. **(E)** Tertiles of uTNC 4 hours after KT showed a graded relationship with the incidence of DGF. Remuzii score **(F)** and IHC score of TNC **(G)** in pre-implantation biopsy of the donor kidney from the DGF recipients and the non-DGF recipients. (*P < 0.05, **P < 0.01, ***P < 0.001).

Serum TNC (sTNC) levels were measured in recipients before, immediately after, and 8 h after kidney transplantation, then every 24 h until postoperative day 4, and then every 48 h until postoperative day 10 (shown in **Figure 1A**). **Figure 1B** shows serial measurements of sTNC levels over this time course. Preoperative sTNC levels were not significantly different between recipients who did and did not develop DGF. sTNC levels rose during the first 24 h postoperatively in all recipients. However, after 24 h, sTNC levels in recipients with DGF continued to rise at the same rate, peaking on postoperative day 4, whereas in recipients without DGF, sTNC levels remained relatively stable for 2–3 days before gradually decreasing, returning to near preoperative levels by postoperative day 10. There was a significant difference in sTNC levels between patients with and without DGF at postoperative day 4 (p=0.0001, shown in **Figure 1B**). Dividing patients into quartiles based on sTNC levels at postoperative day 4 revealed that the highest quartile included the greatest proportion of patients with DGF (7/9, 78%, shown in **Figure 1C**).

Additionally, urinary TNC (uTNC) levels were measured in transplant recipients 4 and 8 h after kidney transplantation, then every 24 h until postoperative day 4, and then every 48 h until postoperative day 10 (shown in **Figure 1A**). **Figure 1D** shows serial measurements of uTNC levels over this time course. In contrast to sTNC levels, uTNC levels were higher in recipients without DGF than in those with DGF and this difference was significant in the early postoperative period (p=0.047 at 4 h and p=0.003 at 8 h postoperatively). Interestingly, the elevated early postoperative uTNC levels in the non-DGF group decreased within 24 h to levels similar to those in the DGF group. Dividing patients into tertiles based on uTNC levels at 4 h postoperatively revealed that as the uTNC levels decreased, the incidence of DGF increased, with the lowest tertile including the highest proportion of patients with DGF (5/6, 83.33%, shown in **Figure 1E**).

### 3.3 TNC staining in pre-implantation graft renal biopsies as a predictor of DGF

To investigate whether donor kidneys provide additional information on the risk of DGF, pre-implantation graft renal biopsies obtained from donor kidneys were analyzed. Hematoxylin and eosin and periodic acid-Schiff staining were performed and analyzed to calculate the Remuzzi score. TNC was detected using immunohistochemistry and semi-quantitative scoring of the staining was carried out. As TNC is synthesized in the endoplasmic reticulum and secreted into the extracellular space, the extent and intensity of positive staining in the interstitium of the kidney cortex were measured.

The results showed no significant differences between renal allografts that went on to exhibit DGF and those that did not, either in the total Remuzzi scores (shown in **Figure 1F**), or in any of the constituent subcategories, including glomerular sclerosis, tubular atrophy, interstitial fibrosis, and degree of narrowing of the small and arteriolar lumens. However, renal allografts that went on to exhibit DGF showed significantly greater TNC staining intensity and a broader positive staining area than those that did not (p=0.004, shown in **Figure 1G**).

### 3.4 Comparing the performance of sTNC levels with conventional biomarkers

**Figure 2:**
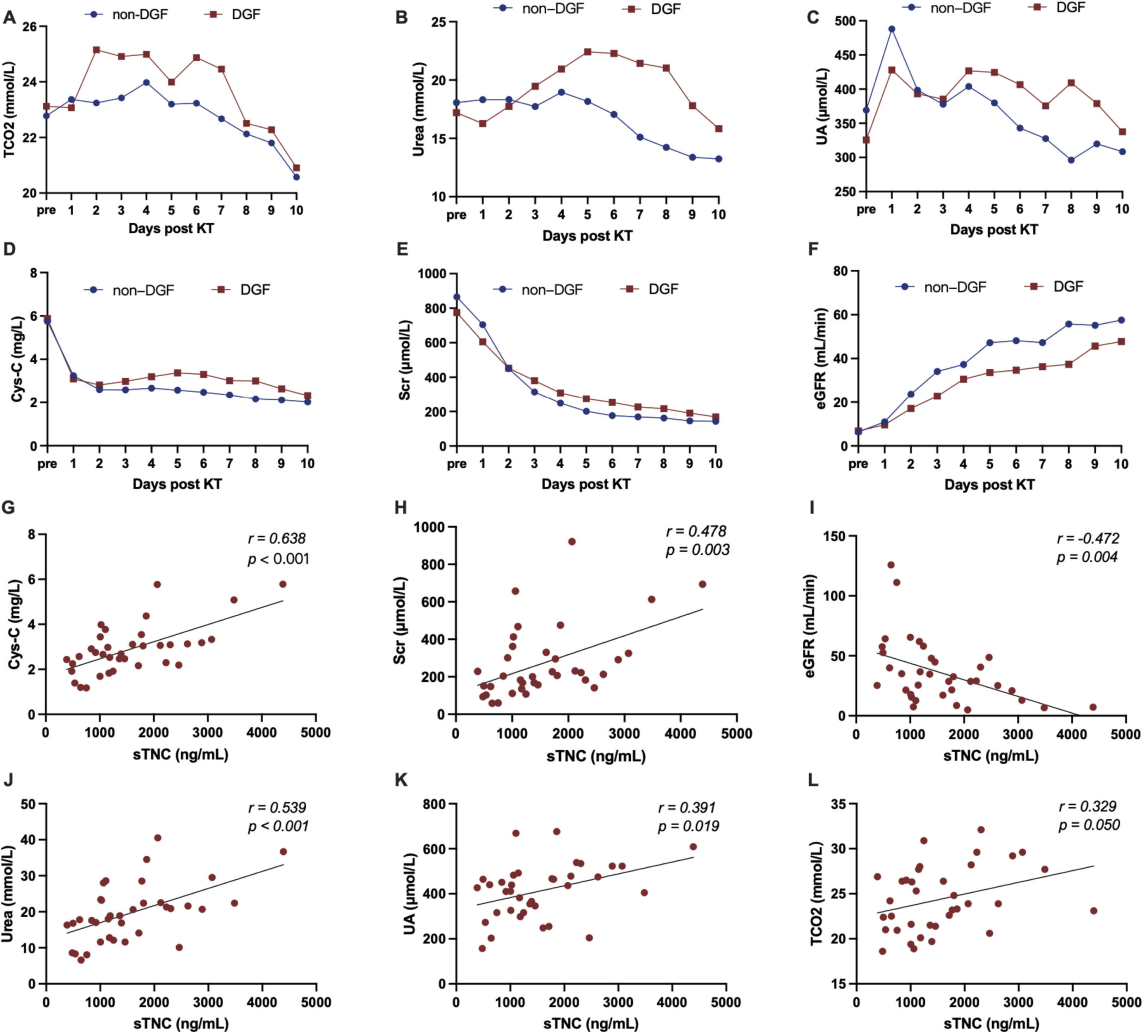
**(A–F)** The level of conventional clinical indicators during the perioperative period of kidney transplantation. **(G–L)** The correlation between sTNC levels and conventional clinical biomarkers on the 4th day after KT.

Traditional renal functional indicators, including total carbon dioxide (TCO_2_), urea, uric acid (UA), cystatin C (Cys-C), and Scr levels, and the eGFR, were also measured during the perioperative kidney transplantation period. The results showed that although TCO_2_, urea, UA, Cys-C, and Scr levels were generally higher, and the eGFR lower, in patients with DGF than in those without DGF, there were no significant differences between the groups (shown in **Figures 2A–F**). Other commonly used clinical indicators, such as white blood cell, neutrophil, and lymphocyte counts, and C-reactive protein, potassium, and sodium ion concentrations also showed no significant differences between the two groups. On postoperative day 4, sTNC levels were significantly correlated with urea, UA, Cys-C, TCO_2_, Scr levels, and the eGFR, supporting the predictive potential of sTNC for renal function (shown in **Figures 2G–L**).

### 3.5 Effect of TNC on prediction of DGF

Univariate logistic regression analysis of baseline data was performed to identify factors predictive of DGF (**Table 2**). Factors with a p-value <0.100, such as sTNC levels at postoperative day 4 (p=0.005), surgical duration (p=0.091), and TNC immunohistochemical staining scores (p=0.009), were included in the multivariate logistic regression analysis. Multivariate logistic regression analysis identified sTNC levels at postoperative day 4 (p=0.039) and TNC immunohistochemical staining scores (p=0.013) as independent risk factors for DGF.

**Table 2:** Results of univariate logistic regression.

Variates	OR (95% CI)	AUC	p value
Age	1.027 (0.969 to 1.093)	0.585	0.384
Cholesterol	1.140 (0.884 to 1.771)	0.508	0.937
Cold ischemia time	0.894 (0.625 to 1.219)	0.537	0.704
Surgical duration	2.637 (1.010 to 9.203)	0.665	0.091
uTNC	0.981 (0.948 to 1.004)	0.653	0.303
sTNC	1.002 (1.001 to 1.003)	0.772	0.005
Cystatin C	1.805 (0.935 to 4.143)	0.611	0.255
Serum creatinine	1.002 (0.999 to 1.006)	0.557	0.558
eGFR	0.983 (0.952 to 1.009)	0.585	0.384
Urea	1.043 (0.960 to 1.144)	0.585	0.384
Uric acid	1.002 (0.997 to 1.008)	0.585	0.384
TCO2	1.099 (0.914 to 1.341)	0.597	0.319
TNC staining scores	1.435 (1.118 to 2.002)	0.756	0.009

We further assessed the effectiveness of combining sTNC levels at postoperative day 4 and TNC immunohistochemical staining scores of pre-implantation graft renal biopsies to predict DGF. Multivariate logistic regression analysis showed statistically significant differences for both sTNC levels at postoperative day 4 and TNC immunohistochemical staining scores (p=0.016 and p=0.025, respectively), with a combined predictive area under the curve (AUC) of 0.861. Specifically, each unit increase in sTNC levels at postoperative day 4 was associated with a 1.002-fold increase in the risk of DGF, whereas each point increase in TNC immunohistochemical staining scores was associated with a 1.441-fold increase in the risk of DGF. When these two biomarkers were added to a clinical risk model comprising recipient age, cholesterol level, donor cold ischemia time, and surgery duration, its ability to predict DGF improved significantly, with the AUC increasing from 0.6790 (95% confidence interval [CI], 0.499–0.859; p=0.067) to 0.932 (95% CI, 0.855–1.000; p<0.0001). These findings indicated that the inclusion of these two biomarkers significantly enhances the accuracy of DGF prediction, helping clinicians identify patients at high risk of DGF and implement appropriate interventions.

## 4 Discussion

The establishment of predictive models for DGF remains a primary goal in transplantation research. This prospective study investigated the potential of TNC as a predictor of DGF.

Despite generally acceptable cold and warm ischemia times in our cohort, the incidence of DGF remained high, highlighting the need for reliable early biomarkers. In this study, sTNC and uTNC levels were simultaneously measured. uTNC levels differed between the groups as early as 4 and 8 h postoperatively, with sTNC levels higher and uTNC levels lower in patients with DGF compared to those without DGF. This inverse relationship likely reflects differences in renal handling and clearance rather than renal production alone. In patients without DGF, the early postoperative diuretic phase may facilitate efficient urinary elimination of TNC, thereby reducing serum levels, lowering renal burden, and promoting early functional recovery. Conversely, in patients with DGF, impaired glomerular filtration and tubular dysfunction may limit TNC excretion, leading to its persistence in the circulation and lower urinary levels.

While renal clearance appears to contribute substantially to this pattern, local TNC production within the injured kidney cannot be fully excluded. Existing literature indicates that uTNC has been detected in various kidney-related pathological conditions, indicating renal involvement, but other tissues may also participate through proteolytic processing and cellular uptake (23). Therefore, changes in serum and urinary TNC after transplantation likely reflect a combination of graft-specific injury, tubular integrity, systemic inflammatory status, and renal clearance efficiency. The transient high expression of uTNC within 8 h postoperatively may additionally indicate a protective role in early functional recovery of the transplanted kidney. Incorporating these considerations helps explain the divergent serum and urinary TNC levels observed in our cohort and underscores the utility of TNC as an early biomarker of graft injury.

Although serum urea is a routinely used marker of renal function, its increment was not significantly associated with DGF in the present study. This finding should be interpreted with caution, as a type II error cannot be completely excluded. However, the association between serum urea and DGF was consistently weak across analyses, in contrast to the robust and reproducible performance of sTNC, suggesting a true difference in predictive utility rather than a purely statistical artifact. Importantly, serum urea and sTNC differ in their biological kinetics and temporal responsiveness. Serum urea is influenced by multiple perioperative factors, including catabolic state, fluid balance, and nutritional status, which may result in rapid and context-dependent fluctuations and limit its ability to reflect early graft dysfunction. In contrast, circulating TNC more directly reflects tissue injury and inflammatory activation, potentially providing a more integrated and temporally informative signal of ongoing graft injury. These biological differences may partly explain the superior early discriminatory performance of sTNC for DGF observed in this study.

Although TNC has been proposed as a sensitive marker of inflammation and tissue injury, donor sTNC and uTNC levels did not show predictive value for early DGF in the present study (p = 0.442, p = 0.758). The biological characteristics of TNC expression may explain this finding. TNC is typically expressed at low levels under physiological conditions but is markedly upregulated during systemic inflammation and tissue repair (15, 16). In the context of brain-dead or circulatory-dead donors, who usually experience severe trauma, hemodynamic instability, and multisystem inflammatory responses prior to organ procurement, circulating and urinary TNC levels are more likely to reflect global systemic injury rather than kidney-specific damage. While donor-derived TNC measurements may lack sufficient specificity to predict post-transplant DGF, recipient-derived TNC levels after transplantation may better capture graft-specific injury and recovery dynamics.

In this study, sTNC upregulation within the early postoperative period (within 24 h) may facilitate the repair of IRI and promote early allograft functional recovery. However, excessive or sustained elevation of sTNC beyond 24 h may reflect more severe tissue stress and IRI-induced injury, thereby exacerbating tubular damage and necrosis and contributing to the development of DGF. This temporal pattern aligns with the concept that transient activation of Wnt/β-catenin signaling—known to be promoted by TNC—supports tissue regeneration following AKI, while prolonged activation leads to sustained injury, podocyte damage, tubular injury, myofibroblast activation, renal fibrosis, and eventual chronic allograft dysfunction (15, 16, 24). In this context, elevated sTNC levels parallel worse biochemical renal function indices not because of dialysis-related clearance effects, but because sTNC serves as an injury-associated biomarker capturing the underlying pathological processes that also lead to impaired solute clearance and delayed functional recovery. Similar findings have been reported by Chen and Fu, demonstrating that early TNC supplementation confers protection against AKI in experimental IRI models, whereas persistently elevated TNC levels promote a profibrotic microenvironment (15, 16). Furthermore, TNC depletion after 10 days was shown to inhibit fibroblast proliferation and attenuate renal fibrosis, thereby delaying chronic kidney disease progression (15, 16). Based on these observations, the unique temporal and spatial regulation of TNC expression identified in our study supports its potential role as an early indicator of DGF.

Given that DGF is pathologically characterized by features of AKI, particularly acute tubular necrosis (25), identifying biomarkers closely associated with these pathological processes may enhance the prediction, evaluation, and management of DGF. In this context, the analysis of pre-implantation graft renal biopsies may provide additional valuable information for perioperative and postoperative clinical decision-making (26, 27). Although the Remuzzi score is widely used to evaluate donor kidney quality and predict prognosis, it has limitations. This study showed that the inclusion of TNC immunohistochemical staining scores effectively improved the pathological assessment of the donor kidney and the prediction of DGF. The early availability of this information is a further advantage, allowing prompt assessment and timely intervention. However, this study was limited by its small sample size and lack of external dataset validation. Future multicenter studies are required to validate the clinical utility of TNC as a biomarker of DGF, along with in-depth mechanistic investigations are also needed.

In conclusion, TNC is a promising new biomarker of DGF. Although further validation is required, early measurement of TNC may assist clinicians in identifying patients at a high risk of DGF after kidney transplantation and in planning and initiating appropriate perioperative management strategies. For high-risk patients, close monitoring of postoperative graft function and proactive intervention measures such as enhanced fluid management, timely use of diuretics, and adjustment of immunosuppressive therapy, should be implemented to reduce the risk of DGF. These patients may also require more frequent follow-up and additional supportive treatments to allow potential complications to be detected and addressed early. For low-risk patients, monitoring intensity can be reduced to standard levels to avoid the unnecessary use of medical resources while maintaining appropriate vigilance to ensure early detection of any abnormalities.

## Data Availability

The original contributions presented in the study are included in the article/supplementary material. Further inquiries can be directed to the corresponding author.
